# Systematics and historical biogeography of the old world butterfly subtribe Mycalesina (Lepidoptera: Nymphalidae: Satyrinae)

**DOI:** 10.1186/s12862-015-0449-3

**Published:** 2015-08-20

**Authors:** Kwaku Aduse-Poku, Oskar Brattström, Ullasa Kodandaramaiah, David C. Lees, Paul M. Brakefield, Niklas Wahlberg

**Affiliations:** Department of Zoology, Radiating Butterflies Group, University of Cambridge, Downing Street, Cambridge, CB2 3EJ UK; School of Biology, Indian Institute of Science Education and Research Thiruvananthapuram (IISER-TVM), CET campus, Sreekaryam, Thiruvananthapuram Kerala 695016 India; Department of Biology, NSG, Laboratory of Genetics, University of Turku, Turku, 20014 Finland

## Abstract

**Background:**

Butterflies of the subtribe Mycalesina have radiated successfully in almost all habitat types in Africa, Madagascar, the Indian subcontinent, Indo-China and Australasia. Studies aimed at understanding the reasons behind the evolutionary success of this spectacular Old World butterfly radiation have been hampered by the lack of a stable phylogeny for the group. Here, we have reconstructed a robust phylogenetic framework for the subtribe using 10 genes from 195 exemplar taxa.

**Results:**

We recovered seven well supported clades within the subtribe corresponding to the five traditional genera (*Lohora*, *Heteropsis, Hallelesis*, *Bicyclus, Mycalesis*), one as recently revised (*Mydosama*) and one newly revised genus (*Culapa*). The phylogenetic relationships of these mycalesine genera have been robustly established for the first time. Within the proposed phylogenetic framework, we estimated the crown age of the subtribe to be 40 Million years ago (Mya) and inferred its ultimate origin to be in Asia. Our results reveal both vicariance and dispersal as factors responsible for the current widespread distribution of the group in the Old World tropics. We inferred that the African continent has been colonized at least twice by Asian mycalesines within the last 26 and 23 Mya. In one possible scenario, an Asian ancestor gave rise to *Heteropsis* on continental Africa, which later dispersed into Madagascar and most likely back colonised Asia. The second colonization of Africa by Asian ancestors resulted in *Hallelesis* and *Bicyclus* on continental Africa, the descendants of which did not colonise other regions but rather diversified only in continental Africa. The genera *Lohora* and *Mydosama* are derivatives of ancestors from continental Asia.

**Conclusion:**

Our proposed time-calibrated phylogeny now provides a solid framework within which we can implement mechanistic studies aimed at unravelling the ecological and evolutionary processes that culminated in the spectacular radiation of mycalesines in the Old World tropics.

**Electronic supplementary material:**

The online version of this article (doi:10.1186/s12862-015-0449-3) contains supplementary material, which is available to authorized users.

## Background

As a subtribe, Mycalesina Reuter, 1896 is considered to be one of the most spectacular evolutionary radiations of butterflies in the world [1]. Butterflies of this subtribe, referred to as mycalesines, are arguably the most cosmopolitan and abundant group of butterflies restricted to the Old World tropics. Consequently, they are used extensively in ecological (e.g. [2–5]) and evolutionary (e.g. [6–9]) research. There are at least 300 currently recognised species belonging to Mycalesina occurring in almost all terrestrial habitat types across all the major paleotropical regions (Africa, Madagascar, Asia and Australasia). However, the factors leading to the evolutionary success of this species-rich paleotropical butterfly group in the World Old tropics are not yet fully understood. Knowledge on where and when important divergences have occurred within the group is vital to our understanding of this spectacular Old World radiation. This primarily requires a robust phylogenetic framework for the entire subtribe, which is currently lacking.

The alpha-taxonomy of this butterfly group is currently fairly well-known and relatively stable (perhaps less so in the group in Asia), compared to at least other species-rich paleotropical groups such as Ypthimina, in the region. However, the higher level taxonomy of Mycalesina is not yet adequately resolved. The current generic circumscriptions of mycalesines have been based on a few morphological characters such as the presence or absence of hairy eyes (interommatidial setae), forewing venation and androconial structures [10–15], which have been considered by some authors (e.g. [16, 17]) as parochial and an inadequate character sets for resolving their systematics. The higher level taxonomic uncertainty is partly attributable to earlier authors dividing the subtribe into African, Malagasy and Asian mycalesines. Although reasons for this geographic division have not been explicit, except perhaps to assume that large water gaps could delimit the hierarchy of nature, subsequent authors have followed the trend of studying the mycalesine fauna at regional levels.

The bulk of the continental African species were separated from all other mycalesines by Condamin [15]. Apart from two species he considered particularly distinct that he placed in the genus *Hallelesis* Condamin, 1961, the rest were placed in *Bicyclus* Kirby, 1871. Although Condamin first introduced *Hallelesis* in 1960, he omitted to assign a type species and so rectified this mistake a year later. Condamin’s revisionary work spanned more than a decade and his comprehensive revision of *Bicyclus* was summarized in an extensive monograph [10]. The genera *Hallelesis* and *Bicyclus* (with two and about 90 currently recognised species, respectively) are distributed almost exclusively on sub-Saharan continental Africa with a single species (*B. anynana*) extending its distributional range to the Comoros and Socotra Islands, both in the Indian Ocean [16, 17]. The Socotran subspecies (*B. anynana socotrana*) was originally placed in the Asian genus *Calysisme* Moore, 1880 despite limited morphological similarities, demonstrating further the entrenched and parochial approach employed by earlier authors in delineating mycalesine groups geographically.

Nearly two and half decades after the publication of Condamin’s work, Lees [13] undertook a similar revisionary treatment of the mycalesines on Madagascar, and arranged them under five pre-existing generic names: *Admiratio* Hemming, 1964, *Masoura* Hemming, 1964, *Henotesia* Butler 1879, *Heteropsis* Westwood, [1850] and *Houlbertia* Oberthür, 1916. However, a subsequent phylogenetic study [18] based on two mitochondrial markers found that most of the Malagasy mycalesine genera were not monophyletic, prompting taxonomic changes of the group on the island. Lees and colleagues [19] followed up and radically revised the taxonomy of mycalesines on the island; synonymising the genus *Houlbertia* and downgrading four pre-existing genera to the status of subgenera under a single genus, *Heteropsis* Westwood, [1850]. At the moment, there are about 75 *Heteropsis* taxa recognised in the Malagasy region, of which about a third are still undescribed [13]. *Heteropsis* occurs on continental Africa as well, with all 13 currently recognised species belonging to the (sub) genus *Henotesia* [20, 21].

The Oriental and Australasian mycalesines have gone through several regional taxonomic revisions. The first major treatment of the group as a whole was made by Moore [12] who divided all Asian mycalesines into 23 genera based primarily on androconial configurations and wing venation. As many as 10 of these new genera were described as monotypic, and this rather extreme splitting was later criticised by other authors (e.g. [22, 23]). Fruhstorfer returned all species to *Mycalesis,* using only two subgenera, but retained *Orsotriaena* Wallengren, 1958 (that had previously been given equal status as all the other genera created by Moore), separate from all other species that were now merged again as subgenus *Mycalesis*. Unfortunately, Fruhstorfer’s work seems to have been somewhat neglected since Moore’s numerous genera remained in use in mainly English texts until they were again demoted by Evans [24] who, just like Fruhstorfer, considered *Orsotriaena* separate, but gave both of Fruhstorfer’s subgenera full generic status. *Orsotriaena* has subsequently been shown in recent molecular studies not be closely related to Mycalesina [25, 26].

Following the taxonomic revision of the group by Evans [24], Moore’s genera [12] were treated as subgenera in some later works. The most important later taxonomic modifications of the Asian Mycalesina were the inclusion of the odd mimetic species *M. drusillodes* (originally described as *Hamadryopsis drusillodes* Oberthür, 1894 with the female in the same work as *Drusillopsis dohertyi*) into *Mycalesis* [27], and the separation of 19 mycalesine species confined to the Sulawesi and Sula islands [14, 28, 29] . These last endemic taxa were treated separately as the genera *Nirvanopsis*, Vane-Wright, 2003 and *Lohora* Moore, 1880, with two and 17 recognised species, respectively. However, a recent molecular study found the two *Nirvanopsis* taxa nested within *Lohora* and thus the former was subsumed within the latter to reflect phylogeny [30]. Kodandaramaiah and colleagues [30] also split the genus *Mycalesis* into two genera, with the clade containing the type species of the genus (*Papilio francisca*) classified as *Mycalesis sensu stricto*, while the other well supported clade was placed under the genus *Mydosama* Moore, 1880 (with *Dasyomma fuscum* Felder & Felder, 1860 as the type species).

The rather turbulent taxonomic history of mycalesines at generic and subgeneric levels has allowed very little progress in our understanding of the interrelationships between the main groups on the different continents. To date, there is no robust phylogenetic framework establishing with appreciable confidence the interrelationships among the six current genera and the many species groups of mycalesines distributed in the different regions of the Old World tropics. Such a framework is crucial to facilitate further studies from the species level to entire radiations, and also to form a stable basis for the higher-level taxonomy of the subtribe. In this study, we have used 10 genes and about 200 exemplar taxa to infer the phylogenetic relationships of species across the entire subtribe Mycalesina. Within the proposed phylogenetic framework, we have estimated times and places of major divergences, and related these with external factors that may have contributed to the success of this subtribe of palaeotropical butterflies.

## Results

### Systematics

The final molecular data matrix comprised 195 taxa representing 185 mycalesine species and 10 related taxa as outgroups. More than 75 % of the sampled exemplar taxa had at least half of the total gene coverage, with the average coverage per taxon being *ca.* 70 % (Additional file 1: Table S1). The resultant alignment consisted of 7735 base pairs of which 46 % and 35 % were variable and parsimony informative sites, respectively. The best partitioning schemes and the optimal evolutionary models for each of the partitioned dataset for the RaxML analyse are listed in Additional file 2: Table S2. The Effective Sample Sizes (ESS) for all the parameters of the different independent Markov Chain Monte Carlo (MCMC) runs in the Bayesian analysis were higher than 200. The Combined ESS for the parameters of the three independent BEAST runs are presented in Additional file 3: Table S3.

**Fig. 1:**
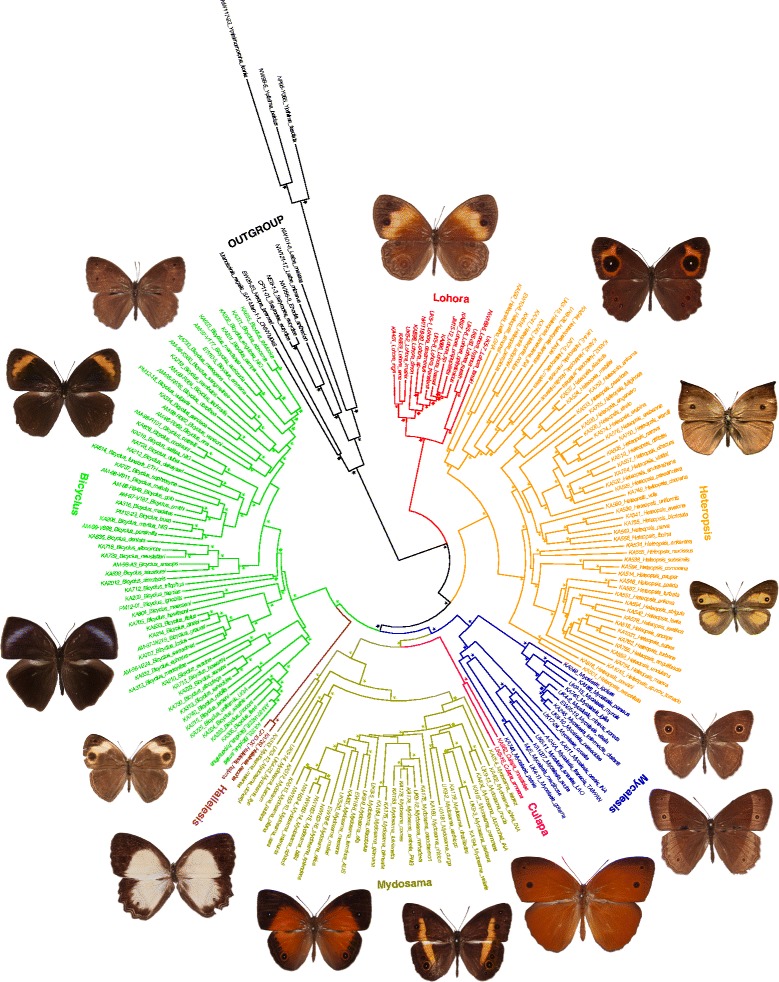
Phylogenetic relationships of the different genera within the subtribe Mycalesina. A (MrBayes) Bayesian phylogeny is shown using the combined 10 genes dataset. An asterisk denotes nodes with posterior probability (PP) more than 0.95. Filled circles are support values (PP) of 0.90-0.94. The colour-coded branches represent the main distinct clades or genera within the subtribe, Mycalesina Reuter, 1896. In a clockwise order starting from the *Lohora* clade, the exemplar images are *Lohora dinon*, *Heteropsis oculus*, *H. drepana*, *H. narcissus*, *Mycalesis igoleta*, *Mycalesis sudra*, *Culapa amoena*, *Mydosama barbara*, *Mydosama messene*, *Hallelesis asochis*, *Bicyclus larseni*, *B. graueri*, *B. matuta* and *B. vansoni*

Our results revealed seven well supported higher clades within Mycalesina (Fig. 1, Additional file 4: Figure S1). Both the Maximum Likelihood (ML) and Bayesian Inference (BI) methods recovered the subtribe Mycalesina as monophyletic with high posterior probabilities (PP) and strong bootstrap supports (BS). Likewise the monophyly of four of the six currently circumscribed genera (*Bicyclus*, *Hallelesis, Mycalesis* and *Lohora*) within Mycalesina were well supported in all the analyses. The Asian mycalesine taxa were recovered as four distinct clades in all the phylogenetic reconstruction methods. One of the Asian clades which for convenience is referred to here as the “Asian *Heteropsis* clade”, includes *Mycalesis oculus, M. adolphei, M. sangaica, M. malsara, M. inopia, M. nicotia/misenus, M. mamerta* and *M. janardana*. This clade was nested within the Afro-Malagasy *Heteropsis* clade with high support in both ML (BS = 95) and BI (PP = 1) methods. The two major Asian clades (*Mycalesis* and *Mydosama*) grouped together but with moderate to high support (PP = 0.99, BS = 56). The topologies were congruent between the different phylogenetic analyses. *Mycalesis* included as its members the type species of the currently circumscribed genus *Mycalesis*, *M. francisca*, and taxa such as *M. sudra*, *M. visala*, *M. gotama, M. patnia, M. mineus, M. oroatis* (see Fig. 1 for full list). Most species of *Mycalesis* were found to be distributed largely on mainland Asia, with the exception being *M. perseus*, which extends its distributional range to the Sundaland, Moluccas and Australasian regions. *Mydosama,* however, comprised taxa such as *M. fuscum, M. sara, M. discobolus, M. itys, M. mucia* and 30 others, which are predominantly distributed on the islands of South-east Asia and Australasia. The last distinct clade comprised of two taxa, *M. mnasicles* and *M. amoena* (Fig. 1, Additional file 4: Figure S1).

The evolutionary relationships between the seven identified higher clades within Mycalesina were largely congruent among the different phylogenetic analyses with moderate to high support (Fig. 1). *Lohora* (together with *Nirvanopsis*) was recovered as the sister clade to all other mycalesines. The two African genera, *Bicyclus* and *Hallelesis*, clustered with high to moderate support values (PP = 0.99, BS = 61 %) as sister clades in all analyses. The *Bicyclus* + *Hallelesis* clade grouped with the two Asian mycalesine clades as sister groups, but this relationship was weakly supported in the ML tree. The *Heteropsis* lineages of the different geographic regions were recovered as well supported monophyletic groups, with the Africa and Asian clades being more closely related than either is to their congenerics on Madagascar. Together, the three continental clades constituted a well-supported *Heteropsis* clade (PP = 1, BS = 100 %) which was sister to other mycalesines, except *Lohora*.

### Diversification times

**Fig. 2:**
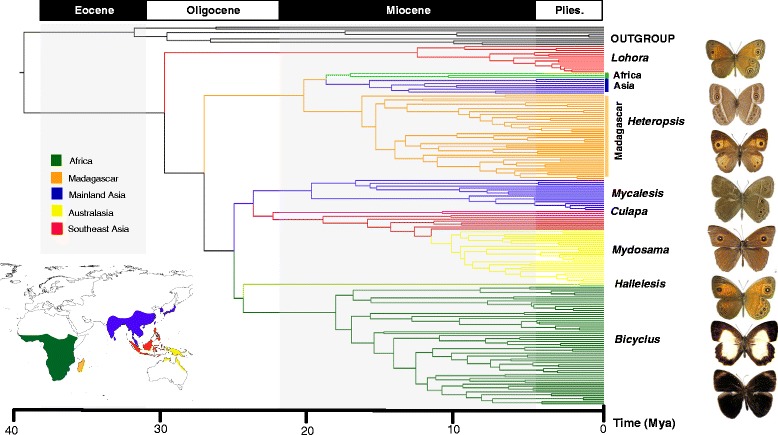
Ultrametric tree showing important times of divergence within mycalesines. The tree is scaled in million years ago (Mya). The insert map is colour-coded to match the present distribution of the exemplar taxa included in our study. Representative images in descending order are *Lohora opthalmicus*, *Heteropsis perspicua*, *H. narcissus*, *Mycalesis perseus*, *Culapa mnasicles*, *Mydosama itys*, *Hallelesis asochis* and *Bicyclus abnormis*

Our divergence time analyses indicate that the common ancestors of the subtribes Mycalesina and Lethina diverged from each other in the Eocene, about 39.8 Mya, with 95 % highest posterior densities (HPD) of 34.2-45.0 Mya (Fig. 2). However, the first major divergence within Mycalesina was not observed until 12 Mya after the Mycalesina-Lethina split. This basal cladogenesis in the mid Oligocene (28 Mya; HPD, 23.8-32.3 Mya) gave rise to the Sulawesi endemic genus, *Lohora.* Subsequent to the split of *Lohora*, the follow-up basal divergences within Mycalesina have been rapid and in tandem, such that by early the Miocene (around 20 Mya), all the currently circumscribed genera had been established. The divergence between the two endemic African genera *Bicyclus* and *Hallelesis* was observed to have occurred around the Oligocene and Miocene boundary, roughly at the same time as the split between the two large Asian clades, *Mycalesis* and *Mydosama*, is suggested to have occurred. Earlier, *Heteropsis* (including the lineages in Africa, Madagascar and Asia) had diverged from the other mycalesines in the Oligocene, around 26 Mya (HPD, 23.4-29.8 Mya). However, the two important divergences within the genus giving rise to the extant lineages on the different continents we estimate to have occurred in the mid Miocene. The Malagasy clade was the first to diverge at around 21 Mya (HPD, 17.2-23.3 Mya). The continental African and the Asian *Heteropsis* clades split about 2.3 Mya later. The onsets of diversifications within most of the genera or higher clades appear to have occurred in the mid Miocene between 20-10 Mya, with the exception of *Lohora* and *Hallelesis. Lohora* as a genus only started diversifying around 11 Mya, despite being the first mycalesine lineage to diverge around 28 Mya. The only two extant species of *Hallelesis* (*H. halyma* and *H. asochis*) are estimated to have diverged only recently, around 1.6 Mya (HPD, 0.9-2.5 Mya).

### Historical biogeography of the subtribe Mycalesina

**Fig. 3:**
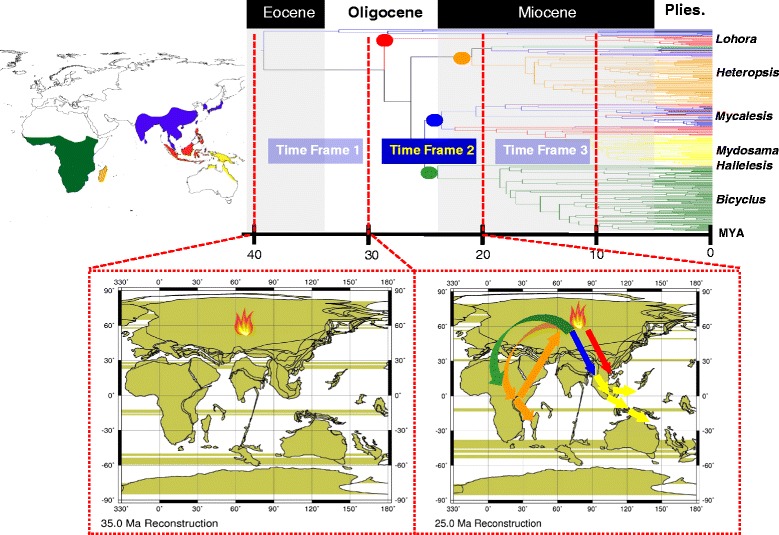
Schematic representation of the reconstructed historical biogeography of mycalesines. Inserts are geographic maps showing the zoogeographic regions colour coded to match the distribution of the exemplar taxa included in our study and a time calibrated phylogeny of the group sliced into three time frames (at 10 Mya intervals) representing the origin of the subtribe, major divergences between, and within the genera, respectively. Arrows are coloured by genus: green (*Bicyclus* and *Hallelesis*), red (*Lohora*), orange (*Heteropsis*) and blue leading to yellow (*Mydosama*). The fire denotes the putative place of origin of the subtribe. Paleomaps were reconstructed using the ODSN Plate Tectonic Reconstruction Service (http://www.odsn.de/odsn/services/paleomap/paleomap.html). The tree is scaled in millions of years ago (Mya)

Our ancestral area reconstruction analyses suggest that the common ancestors of the subtribes Mycalesina and Lethina were most likely distributed in present-day Asia, making Asia the ultimate origin of the subtribe Mycalesina. Both reconstruction methods identified multiple events of vicariance and dispersal in the historical biogeography of mycalesines. We infer from our results the following scenario. Africa has been colonized at least twice by Asian ancestors. The first observed dispersal event from Asia to Africa resulted in the *Heteropsis* clades on mainland Africa and Madagascar. The second colonisation of Africa by Asian ancestors gave rise to the two currently circumscribed endemic African genera, *Hallelesis* and *Bicyclus*. The split between *Hallelesis* and *Bicyclus* most likely occurred in Africa. The mycalesine fauna of the Wallacean and Australasian regions (*Lohora* and *Mydosama*) were derived from ancestors from mainland Asia (Fig. 3).

## Discussion

### Systematic implications

This study represents the most comprehensive molecular investigation of the phylogeny of the subtribe Mycalesina. Before the present study, only one molecular study [30] has made a coherent attempt to classify the entire subtribe Mycalesina. The two other molecular studies of mycalesines [18, 31] focused on the group at regional levels. Kodandaramaiah and colleagues [30] used 125 exemplar taxa (of 90 species) and three genes totalling 3139 base pairs (bp) in length in their phylogenetic reconstructions. In this study we have used 195 taxa and 10 gene regions (~7735 bp). The increase in both the number of taxa and genes in the present study appears to have helped to resolve most of the previously unresolved relationships within Mycalesina. Against the backdrop of the taxonomic confusion surrounding the group, especially its members in Asia and Madagascar, we hope that this study will put to rest the frequent taxonomic fluxes within the group. We have established, for the first time with good confidence levels, the evolutionary relationships between the different mycalesine groups of the different palaeotropical regions.

Our phylogenetic hypothesis largely corroborates earlier findings by Kodandaramaiah et al. [30]. For instance, both studies confirmed the monophyly of the subtribe Mycalesina and three higher clades or genera; *Bicyclus*, *Hallelesis* and *Lohora*, with relatively higher nodal supports in the present study. Again, the circumscribed ‘catch-all’ Asian genus *Mycalesis* was recovered as a polyphyletic group just as in Kodandaramaiah and colleagues [30]. However, we recovered the sampled *Mycalesis* (*sensu lato*) taxa as three and not the four distinct clades previously reported. Kodandaramaiah et al. [30] found two Asian (*Heteropsis*) clades, (*Heteropsis adolphei*) and (*H. sangaica* + *H. mamerta* + *H. malsara* + *H. janardana*), nested within the Afro-Malagasy clade but in a polyphyletic fashion. We recovered all the Asian *Heteropsis* taxa as a single well-supported clade inside the Afro-Malagasy clade.

### *Mycalesis*, *Mydosama* and *Culapa*

As clearly shown in the results, most of the Asian and Australasian taxa segregated into two major clades, supporting an earlier suggestion by Kodandaramaiah and colleagues [30] to split the currently circumscribed *Mycalesis* into at least two genera. One clade, referred to here and in Kodandaramaiah et al. [30] as *Mycalesis sensu stricto* contains the type species of the genus (*Papilio francisca*) and 36 other taxa occurring predominately on subcontinental India, Indo-China and marginally into Sundaland. Interestingly, many taxa of this clade that occur in Sundaland are only represented in the Western part (*i.e.,* Peninsular Malaysia), closest to the Asia/Indo-China mainland. We support an earlier proposal to classify this clade as *Mycalesis sensu stricto*. The other clade, referred to here and in Kodandaramaiah et al. [30] as *Mydosama*, is nearly allopatric with *Mycalesis*. Taxa of this clade largely include mycalesine species distributed in Sundaland, the Philippines and the Australasian regions. Kodandaramaiah et al. [30] suggested this clade be transferred to *Mydosama*, Moore 1880, which has the type species, *M. fuscum* Felder and Felder 1860. However, with the inclusion of *M. mnasicles* as part of this clade in our reconstruction, the name *Mydosama* may not be appropriate (potentially junior), as it post-dates the name *Culapa* Moore, 1878, which has *Dasyomma mnasicles* as the type species. From our phylogeny, the taxa *Mycalesis mnasicles* and *M. amoena* are retrieved as sister to all taxa of *Mydosama. M. mnasicles* is currently distributed in Indo-China and marginally into Sundaland. *M. amoena* is however restricted in distribution range to forests in Borneo.

Given the unique morphology of *M. mnasicles* and *M. amoena*, coupled with the moderate to weak support in the ML topology (BS = 51 %, PP = 0.98) for their sister-relationship with the rest of *Mydosama*, we split off the taxa *M. mnasicles* and *M. amoena* and classify them as a separate genus. With this approach, both *Culapa* and *Mydosama* can be brought into use while promoting stability within this rather complex species group. The taxon sampling in Asia is relatively low in our study compared to that in Africa and Madagascar. Thus we have sampled ~68 % of all recognised mycalesines in Asia in the current study compared to ~82 % in the Afro-Malagasy region. It would therefore not be surprising if increased sampling in Asia revealed more members of the genus *Culapa*, or even additional distinct lineages or genera within mycalesines in the region. *Culapa* was described as a monotypic genus by Moore [12]. But as has been shown in our study, many of the original taxa described by Moore on the basis of adult morphology as monotypic genera (e.g. *M. maianeas* as *Satoa* Moore, 1880, *M. orseis* as *Suralaya* Moore, 1880, *M. adolphei* as *Telinga* Moore, 1880, *M. oroatis* as *Loesa* Moore, 1880) are not entirely accurate; rather they have closely related sister species. It is worth noting that Moore’s classification was solely based on androconia (or scent-producing organs as he called them) and wing shape. Previous studies have shown the problems such as homoplasy that result in the use of a few morphological characters that are not under relatively neutral selection in such classifications [13, 32]. We are of the view that a detailed molecular investigation of the mycalesines in Asia will help refine our knowledge on their rather complex morphology and, ultimately, their systematics.

### *Heteropsis*

This is the only genus that has representations in all the major paleotropical regions, according to the phylogenetic hypothesis presented in this study. Our proposed framework provides support for monophyly of each of the *Heteropsis* species groups on the different continents or islands. Whilst the numbers of *Heteropsis* species in the Malagasy region and mainland Africa is estimated at around 75 and 13 respectively, the richness in Asia is unknown because mycalesines in this region have also been treated broadly as *Mycalesis* in which a large number of names exist for different island and mainland populations. Kodandaramaiah et al. [30] recovered five *Mycalesis* species (treated here as *H. adolphei, H. sangaica, H. mamerta, H. malsara,* and *H. janardana*) as belonging to the *Heteropsis* clade, one of which, *H. adolphei*, has earlier been suspected to be within this genus based on genital morphology [13]. In the present study we recovered, in addition to the above five taxa, three additional Asian taxa (*H. oculus, H. inopia,* and *H. nicotia/misenus*) clustering within this largely Indian *Heteropsis* clade. This well-supported Indian *Heteropsis* clade (BS = 99, PP = 1) was nested within the Afro-Malagasy *Heteropsis* clade.

Two of the sampled *Mycalesis* taxa (here treated as Asian *Heteropsis*), *H. janardana* and *H. sangaica*, have long been considered as a separate species-group since Moore [12] described them as belonging to the genus *Martanda* Moore, 1880, together with the then taxon *M. megamede* Hewitson, [1862] (which was later found to be a junior synonym of *M. janardana*). With the exception of *H. oculus* and *H. adolphei*, all the remaining sampled Asian *Heteropsis* are categorised according to Aoki et al. [28] as belonging to one informal subgeneric unit called species-group 3. It is very likely from morphology that, the (molecularly) unsampled members of Aoki and colleagues’ [28] species group 3 (*M. heri, M. mestra, M. misenus, M. annamitica, M. lepcha*) belong the Asian *Heteropsis* clade. However, we are uncertain in the case of *M. suaveolens*, that was also included by Aoki et al. [28] in this group, as it lacks the typical irrorated pattern of the ventral wing surfaces of species in this group. *H. oculus* was similarly misplaced in the species group 2 of Aoki et al. [28], together with *Mycalesis* taxa such as *M. perseus, M. mineus, M. visala* and *M. perseoides*. The latter were recovered as part of clade *Mycalesis* I or *Mycalesis sensu stricto* [30]. We also confirm that *H. oculus* is most closely related to *Satyrus adolphei* (type species of *Telinga,* Moore, 1880) which occurs on the other side of the Palghat Gap [4]. At the moment, *H. oculus* (and by association *H. adolphei*) seems to be the only Asian *Heteropsis* taxon ‘wrongly’ placed in the subgeneric classification of Aoki et al. [28]. However, it is still unclear how many of the unsampled *Mycalesis* species in the different species-groups of Aoki et al. [28] actually belong to the Asian *Heteropsis* clade. Here, we reiterate the importance of a more comprehensive molecular and morphological study of the mycalesines in this region.

According to our phylogenetic hypothesis, the relationships among the Afro-Malagasy *Heteropsis* species were not consistent with subgeneric classification proposed in Lees [13] and Lees et al. [19]. For instance, the hierarchically reciprocal monophyly of each *Heteropsis* clade on the different continents in the present study suggests that the currently circumscribed subgenera *Telinga* and *Henotesia*, which include taxa occurring in and outside the Malagasy region in some treatments [21–23] are incorrect. About a third of the total mycalesine fauna in Madagascar are currently being described as new (D. C. Lees, *submitted*). It will be useful to include samples of these newly described taxa in a detailed molecular study that holistically explores the intricate evolutionary history of this widely distributed butterfly group across the Old World tropics.

### *Lohora*

As shown in our results, the two species of the subsumed genus *Nirvanopsis* Vane-Wright, 2003 (*N. susah* and *N. hypnus*) are clearly nested within *Lohora* as was also found by Kodandaramaiah et al. [30]. The relationship among *Lohora* species is highly consistent with the three recognised subgeneric divisions [14] which could therefore be retained if required. A clade comprising *L. erna, L. imitatrix* and *L. inga* in our tree represented the subgenus *Physcon* de Nicéville, 1898, which has five other taxa (*L. decipiens, L. umbrosa, L. deianira, L. deianirina* and *L. pandaea*) that were not sampled in our study. Six of the seven described species of the nominate subgenus *Lohora* Moore, 1880 clustered in our tree as a well-supported clade. The monotypic subgenus *Pseudomycalesis* Tsukada and Nishiyama, 1979, currently consisting of only *L. tanuki*, should however be redefined or expanded to include the two taxa of the junior genus *Nirvanopsis,* to reflect our phylogeny. This proposed redefinition of *Pseudomycalesis* is also supported by the superficial similarities in their genitalic morphology and eye-spot patterns on the hind wings [14].

### *Bicyclus* and *Hallelesis*

The African genera, *Bicyclus* and *Hallelesis*, are the only mycalesines that lack conspicuous hairs on their compound eyes. From our proposed phylogenetic framework, it appears that this interommatidial setal trait was lost in Africa, where these genera are inferred to have split, and since no other naked eye mycalesines are known in Africa. Members of *Hallelesis* and *Bicyclus* were regarded as a single genus until Condamin [33] separated them based on the single character of the presence of long scent-producing hair-tufts in the male genitalia of the former. A stronger and more convincing justification for the generic separation is provided by the present and by past studies based on molecules [30, 31], as well as by morphological studies of immature stages [34] of African mycalesines.

Nevertheless, the phylogenetic position of these two African endemic clades in the entire mycalesine radiation has not been resolved previously. Although *Bicyclus* and *Hallelesis* are similar in terms of life history traits [34] and are often seen flying together in the wild [35], previous molecular studies (albeit with weak nodal supports) found the latter genus to be more closely related to either *Heteropsis* [18, 31], or to the Oriental *Mycalesis* [30]. The relationship of *Bicyclus* to other mycalesines has also never been strongly established. Kodandaramaiah and colleagues [30] without strong support, retrieved *Bicyclus* as the sister taxon to all mycalesines, a hypothesis that has fundamental implications for the origin of the entire subtribe. Including just two exemplar taxa of *Bicyclus* in their study of mycalesines in Madagascar, Torres et al. [18] recovered it without strong support as a nested clade within the Malagasy *Heteropsis*. The latter authors, however, admitted this relationship might be an artefact of their poor taxon sampling of *Bicyclus*. Based on our present study, we consider *Hallelesis* and *Bicyclus* as sister clades with a common ancestor derived from Asia. *Hallelesis*, that we still consider as a different genus, diverged long (~24 Mya) before the diversification of *Bicyclus* into its extant taxa. The similarity in life history traits in the immature stages [34] and the coexistence of the adults of *Hallelesis* and taxa of the basal clade of *Bicyclus* (*evadne* species-group) in the wild [35] further strengthens our hypothesis that the two genera are sister taxa.

In general, the relationships between the major mycalesine lineages or genera are reasonably stable and congruent among the different methods. In the light of a relatively well resolved backbone topology in the current phylogenetic hypothesis, we next discuss the implications of these findings for our understanding of the historical biogeography of Mycalesina.

### Historical biogeography

Our ancestral range estimation methods clearly point to present-day Asia as the area of origin of the subtribe Mycalesina. We hypothesise that the common ancestors of Mycalesina and Lethina were distributed in Eurasia during the Oligocene until they split around 38 Mya. This hypothesis is further strengthened by the discovery of a fossil (*Lethe corbieri*) of the mycalesine putative sister group Lethina [36] in Oligocene deposits of south eastern France [37]. A vicariance event is hypothesised to be responsible for the first split between the Moluccan *Lohora* and the rest of Mycalesina, although oceanic dispersal is not completely ruled out [38]. Until the Eocene epoch, all islands in the western Pacific Ocean (including those on the Sundaland and Asian islands) were putatively part of the ‘supercontinent’ Asia [38–41]. This contiguous landmass is believed to have facilitated faunal movements and gene flow among different populations in the region at that time.

However, the gradual formation of the Makassar Strait (between present-day Borneo and Sulawesi) in the Eocene is thought to have severed the land connection [38, 40, 42]. Available paleontological evidence suggests that by the middle of the Miocene, most of the land of proto-Sulawesi had disappeared below sea-level, leaving only a small part of the island until the mid-Miocene [42, 43]. The Makassar Strait is implicated to have caused a disjunction and a physical barrier to biotic exchanges or gene flow between the common ancestors of all extant mycalesines on the mainland Asian and Sulawesi at the time. This implied Paleogene vicariance event is not only peculiar to the historical biogeography of *Lohora*. In their comprehensive review of butterflies on Sulawesi, Vane-Wright and de Jong [29] found an exceptionally high number of endemics at the species level (>40 %). This was in stark contrast to the level of endemism at the genus level. Almost all the genera (>95 %) on the island have representatives on the mainland Asia, with no special relationship with Borneo which is just 120 km away from Sulawesi [29, 44]. This observation suggests that most of the butterflies on Sulawesi and its satellite islands might have resulted from an older vicariance or from isolation events, rather than recent dispersals from closer islands or landmasses to the island.

Although these authors [29, 44] found signatures of an older biogeographic event in Sulawesi butterflies, they could not explicitly implicate vicariance as a plausible explanation of what they describe as the “basic puzzle of Sulawesi butterfly fauna” because of the absence of a time-calibrated phylogeny for most species groups at the time. The present study supports the case of a possible Paleogene vicariance event resulting in the Sulawesi endemic mycalesine group *Lohora*. The divergence of *Lohora* from the other mycalesines apparently occurred around 28 Mya (Fig. 2), although the genus only began to diversify some 11 Mya (HPD, 8.3-13.25 Mya), roughly when Sulawesi is thought to have started to expand its land mass. Currently, there are 19 described *Lohora* (sensu *lato*) species (two of which were earlier placed in *Nirvanopsis*) and all are confined to forests of differing types and bioclimatic conditions in Sulawesi [14].

A single species, *L. susah,* is restricted to Taliabu, a Pleistocene landbridge island east of the Sulas [45]. Müller and Beheregaray [46] report a similar time of divergence (ca. 26 Mya) between the Sulawesi lacewing butterflies *Cethosia* and their congenerics on mainland Asia. A similarly older vicariant event is implicated in the split between the *Charaxes* butterflies on the Wallacea and those in other regions in Asia [47]. Some recent molecular phylogenetic studies of Sulawesi taxa such as *Chitaura* grasshoppers [48], wood-feeding cockroaches [49] and mite harvestmen [50] identified Asia as their origin and suggested vicariance as the likely mechanism for the current distribution of the taxa on the island. It remains to be seen whether similar results will be found in other more species rich groups on the island. The only endemic *Mycalesis* species in Sulawesi, *M. itys*, is estimated to have diverged from its sister group in the Miocene *ca.* 14 Mya.

Kodandaramaiah and his colleagues [30] postulated that there has been at least one dispersal event between Africa and Asia. However, they could not infer the direction of such colonisation event due to their inability to distinguish between hypotheses of African and Asian origin for the subtribe, combined with a poorly supported basal relationship in their phylogenetic hypothesis. With the well resolved basal relationships in the current study, we infer at least two events of dispersal of ancestors from Asia to Africa and a later back colonisation of Asia by African ancestors. These dispersal events are dated between 26 and 19 Mya and coincide with the period when Afro-Arabia and Eurasia putatively collided around the Arabian Peninsula [51, 52]. The collision of the Afro-Arabian plates with Eurasia, in the early Miocene, caused the emergence of a terrestrial corridor, called the "Gomphotherium" Landbridge [53]. This putative land bridge is believed to have facilitated faunal exchanges between Africa and Eurasia in the Miocene, as reported in the butterflies *Junonia* [54] and *Charaxes* [55], as well as mammals [52, 56], reptiles [57, 58] and many other organisms. We surmise that the ancestors of mycalesines used this corridor which is thought to have been forest-covered in the Miocene [60]. The subsequent separation of Afro-Arabia and the Indian subcontinent, following the marine connections between the Mediterranean Sea, Indian Ocean and the Paratethys in the mid-Miocene [54] may have partitioned the distribution of mycalesines between the two continents.

The first colonisation event by Asian ancestors to Africa is inferred to have given rise to *Heteropsis* on mainland Africa, which probably dispersed into Madagascar via oceanic dispersal. The ancestral area analyses however, suggest Asia, Africa or both as the origin of the Malagasy *Heteropsis*. The lack of resolution on the origin of Malagasy *Heteropsis* is largely due to the well supported and reciprocal monophyly of the Asian and African *Heteropsis* clades. If Madagascar was indeed colonised from Africa it might be more closely related to some as yet unsampled lineage there. Perhaps including more exemplar taxa of currently unsampled African and Asian taxa, could help refine our understanding on the origin, although a range-constrained analysis in LAGRANGE suggested Africa as the most likely source of Malagasy *Heteropsis*. The second colonisation of Africa by Asian mycalesines is also estimated to have happened in the Oligocene. This event is inferred to have resulted in the African genera *Hallelesis* and *Bicyclus*, neither of which could back-colonise Asia nor disperse successfully to Madagascar. *B. anynana* is found on the island of Grande Comoro and on Socotra, but these are recent colonisation events which have not yet resulted in local speciation.

All the ancestral area reconstruction methods identified Asia as the area of origin for *Mycalesis, Culapa* and *Mydosama*. The splits between these Asian clades are estimated to have occurred at the Oligocene-Miocene boundary (23 Mya). According to our reconstruction, the most recent common ancestors of the resurrected genus *Mydosama* were most likely distributed on Borneo and other Southeast islands such as Sulawesi and Philippines that fractured off mainland Asia between the Oligocene and Miocene. The extant species of *Mydosama* mainly occur on the Wallacean and Australasian regions. The age of split between the Wallacean and Australasian species groups is estimated at 11 Mya. This time of divergence corresponds, or immediately follows, the orogeny of New Guinea and other neighbouring islands. Dispersal into new regions created new ecological opportunities which presumably flattened the adaptive landscape, facilitating a sudden burst in the diversification [59, 60].

## Conclusion

The current study presents the most extensive molecular phylogenetic species-level analysis of mycalesines to date. We have substantially clarified the taxonomy of the group in this study and laid the path towards stabilising the frequent higher-level taxonomic changes associated with the group. Asia is identified as the ultimate area of origin of the subtribe Mycalesina and the crown age of the group is estimated as 40 Mya. We have also established, with strong support, the phylogenetic relationships of the different mycalesine groups across the Old World tropics and proposed the most robust narrative of the biogeographic history of the group to date. The current study paves the way for studies aimed at understanding the fundamental evolutionary mechanisms that operate on large temporal and spatial scales in species radiations. Thus, our time-calibrated tree sets the stage for comparative analyses of the tempo and mode of diversification of the different parallel radiations within the subtribe*.* Our proposed phylogeny serves as a guide for future studies aiming to extrapolate the numerous population level studies within the model species, *Bicyclus anynana* to closely related taxa. Researchers are now in the position to unravel the causes of the spectacular adaptive radiation of mycalesines in the Old World tropics.

Based largely on the molecular evidence provided in this study and some morphological evidence we recommend the following generic classification within the subtribe Mycalesina Reuter, 1896.

Genus *Lohora* Moore 1880. Type species, by original designation: *Mycalesis dexamenus* Hewitson, 1862.

Genus *Heteropsis* Westwood, 1850. Type species, by original designation: *Heteropsis drepana* Westwood, [1850].

Genus *Mycalesis* Hübner, [1818]. Type species, designation: *Papilio francisca* Stoll, [1780].

Genus *Mydosama* Moore, 1880. Type species (replacement name of *Dasyomma* C. & R. Felder, 1860, preocc): *Dasyomma fuscum* C. & R. Felder, 1860.

Genus *Culapa* Moore, 1878. Type species, by original designation: *Mycalesis mnasicles* Hewitson*,* [1864].

Genus *Bicyclus* Kirby, 1871. Type species (replacement name for *Idiomorphus* Doumet, 1861, preocc.): *Idiomorphus hewitsoni* Doumet, 1861.

Genus *Hallelesis* Condamin, 1961. Type species, by original designation: *Mycalesis asochis* Hewitson, 1866.

## Methods

### Taxon sampling

A total of 185 samples representing all current genera and almost all species groups of Mycalesina across their distributional range were collected either by the authors during field expeditions between 2011 and 2014 or by numerous collaborators (see Acknowledgements). Noticeable exceptions are a few species that might well form monospecific groups but for which useful sequences could not be obtained (*M. aramis, M. nala, M. suaveolens,* and *M unica*). Samples for DNA extraction were usually one or two legs, and in a few cases, thoracic tissue of dried mounted vouchers or ethanol-preserved specimens. As outgroups, 10 exemplar taxa were carefully selected using the most recent comprehensive phylogeny of the family Nymphalidae [61]. Genomic DNA was extracted using the Qiagen DNEasy extraction kit, following the guided protocol by the manufacturer. A total of ten molecular markers; one mitochondrial (cytochrome c oxidase subunit I, CO1) and nine nuclear (carbamoylphosphate synthetase domain protein, CAD; Ribosomal Protein S5, RpS5; Ribosomal Protein S2, RpS2; wingless, wgl; cytosolic malate dehydrogenase, MDH; glyceraldehyde-3-phosphate dehydrogenase, GAPDH; Elongation factor 1 alpha, EF-1α; and Arginine Kinase, ArgKin and Isocitrate dehydrogenase, IDH) gene regions were amplified and sequenced for each of the exemplar taxa using primer-pairs obtained from Wahlberg and Wheat [62].

Successful amplicons were cleaned with EXO-SAPIT (Affymetrix) and sent to Macrogen Services (Amsterdam) for Sanger sequencing. DNA sequences and, where possible, aliquots of DNA extracts used in previous mycalesine studies (e.g. Monteiro and Pierce , Kodandaramaiah et al. [31]) were also obtained from the authors and included in the present study. Nucleotide sequence alignment was done by eye using Bioedit [65]. Sequences were then managed and datasets constructed using Voseq v1.7.4 [66]. The software MEGA v6 [67] was used to assess the properties of the sequences of individual genes and the multi-gene concatenated sequence matrix.

### Phylogenetic inference

To minimise the effect of saturation and also improve phylogenetic resolution of our multi-gene dataset, PartitionFinder v1.1.1 [63] was employed to select the optimal gene partitioning schemes and the best-fit model of nucleotide substitution for each partitioned dataset, under the Bayesian Information Criterion (BIC). To check for the degree of congruence among the different markers, phylogenetic analyses were done first separately for each gene (producing gene trees) and later for all the ten genes combined, but partitioned by the optimal gene partitioning scheme suggested by PartitionFinder analyses. Phylogenetic inference analyses were carried out using both Maximum likelihood (ML) and Bayesian Inference (BI) methods.

Maximum likelihood phylogenetic inference analyses was implemented in RAxML-HPC2 v8.0.24, on the CIPRES Science Gateway v3.3 [64], using the partition scheme from the PartitionFinder analysis (Additional file 2: Table S2), under the GTRCAT model for the rapid bootstrapping phase, and GTRGAMMA for the final best scoring ML tree. For bootstrapping, we performed 1000 Maximum Likelihood (ML) pseudo-replicates analyses. Bootstrapping was performed under auto Majority Rule Criterion (autoMRE). BI was carried out using Markov Chain Monte Carlo (MCMC) randomisation in MrBayes v3.2 [65]. We used reversible-jump MCMC to allow for sampling across the entire substitution rate model opposed to specifying one substitution model suggested by PartitionFinder in this analysis. Two parallel runs of four chains (3 heated and 1 cold) were performed for 50 million generations, with sampling done at every 5000^th^ generation. The software Tracer v1.6 [66] was used to inspect the sample sizes of the parameters used in the BI and also check for the convergences or otherwise of the parallel MCMC runs.

### Estimating times of divergence

Times of divergence within Mycalesina were estimated using a Bayesian MCMC approach in BEAST 2, v2.1.2 [67]. Tree calibrations were done using two secondary age calibration points from Wahlberg et al. [61] and one fossil, *Lethe corbieri*, found in the Oligocene deposits of southeast France [37]. It is important to point out that these two approaches are not entirely independent as Wahlberg et al. [61] used *Lethe corbieri* and six other fossils in their divergence-time estimation. Based on Wahlberg et al. [61], the stem age of the subtribe Mycalesina was constrained to be 40 ± 5.5 Million Years Ago (Mya) with a normal distribution prior between the minimum and maximum age bounds. The second calibration point was the crown age of all mycalesines, excluding *Lohora*, and this was constrained to be 26.6 ± 4.5 Mya. It is worth noting that Wahlberg et al. [61] did not include an exemplar taxon of *Lohora* in their study. *Lohora* was recovered as sister to all other mycalesines in our estimate of phylogenetic relationships. Using the estimated age of the *Lethe corbieri* fossil, the minimum age (or offset) of the divergence between *Lethe* and its sister group was constrained at 25 Mya, under an exponential distribution prior.

We implemented a birth-death process with an uncorrelated log-normal distribution model for lineage substitution rate variation. The summarised tree produced using MrBayes was used as the starting tree in the XML file generated using BEAUTi (which is part of the BEAST package). The MCMC chain was run for 50 million generations, with 2 independent runs. The resultant BEAST log files were viewed in Tracer v1.6 [66] to inspect ESS of the parameters and points of convergence. With a threshold of 25 % burn-in, all post burn-in trees from the 4 independent runs were combined using the software LogCombiner v2.1.2 [67]. TreeAnnotator v2.1.2 [67] was used to summarise information (*i.e.*, nodal posterior probabilities, posterior estimates and highest posterior density, HPD limits) from the individual post burn-in trees onto a single Maximum Clade Credibility (MCC) tree. The summarised information were visualised on the MCC tree using FigTree v1.4 (http://tree.bio.ed.ac.uk/software/figtree/). To assess the impact of different tree-shape and clock priors on our results [68], we repeated the analysis with yule and calibrated-yule models of speciation, under both normal and uniform distribution priors between the minimum and maximum age bounds calibration points. There was no significant difference among the results (Additional files 5: Table S4).

### Ancestral range estimation or reconstruction

Ancestral range estimation or reconstruction analyses were done using 100 randomly selected ultrametric trees generated from the BEAST analyses. Ancestral state reconstruction or estimation was done using both as a dispersal-extinction-cladogenesis (DEC) analysis in LAGRANGE [69, 70] and S-DIVA [71]. Both analyses were implemented in RASP [72] and required ultrametric trees and the present distribution of the extant taxa as input files.

Following Proches and Ramdhani [73], the current distributional range of mycalesines was divided into five zoogeographical clusters: 1. Afrotropics (which denotes mainland Africa, south of the Sahara); 2. Malagasy Region (encompasses Madagascar and the surrounding Mascarene islands in the Indian Ocean); 3. Continental Asia (includes the Indian subcontinent, Indo-China); 4. Wallacea (archipelagic transitional zone between Indo-Malaysian, Australian Regions and New Guinea, which includes Sulawesi, Philippines and Moluccas). 5. Australasian Region (covers New Guinea, Solomon Islands archipelago, tropical Australia), Using the present distribution of the extant taxa, each exemplar taxon in the tree was categorised into one or more of the five defined zoogeographic regions.

DEC analyses were carried under two scenarios; (1) unconstrained (where dispersal among all possible pairwise geographic areas are permitted) and (2) constrained (where we defined, *a priori*, the dispersal probability and area-connectivity between geographic areas, using our knowledge of the system). The connectivity matrix for the DEC analyses are in Additional file 6: Table S5. Probabilities to disperse were set to 1 for pairs of areas separated by at least 400 km of water (e.g. Africa and Madagascar, Africa and Asia, Asian and Wallacea, Wallacea and Australasian Region), to 0.1 when two areas were separated by water of a distance less than 3000 km (e.g. Asia and Madagascar), 0.001 and 0.0000001 for long-distance dispersal and highly unlikely scenarios, respectively (e.g. Madagascar and Wallacea, Africa and New Guinea).

### Ethical approval

The funders of the project, European Research Council (ERC) approved the ethics and methods employed in the conduct of the study.

### Availability of supporting data

The data sets supporting the results of this article will be made available in the TreeBASE repository, in http://treebase.org/treebase-web/home.html. Sequences will be submitted to Genbank with accession numbers made available in the final manuscript.

## Additional files

Additional file 1: Table S1. List of exemplar mycalesines taxa included in the study and the gene regions amplified and sequenced per taxon. “x” denotes a case where a gene region was successfully amplified and sequenced for the taxon. Gene regions (COI, Ef1a, MDH, CAD) more than 800 base pairs long were amplified in two parts. (XLSX 35 kb) Additional file 2: Table S2. The PartitionFinder estimated best-fit partitioning schemes and models of molecular evolution of Mycalesina data matrix used in the RaxML tree estimation analysis. The best model was selected under a Bayesian Information Criterion (BIC). (DOCX 20 kb) Additional file 3: Table S3. The estimated mean values and Effective Sample Sizes of parameters used in the BEAST time of divergence analyses. The figures are combined values of three independent BEAST Monte Carlo Markov Chain analyses, each ran for 50 Million generations. (XLSX 14 kb) Additional file 4: Figure S1. Phylogenetic relationships of the different genera within the subtribe Mycalesina estimated using RaxML maximum likelihood method. A consensus phylogeny is shown using the combined 10 genes dataset. The numbers besides the nodes are the bootstrap support values. (PDF 82 kb) Additional file 5: Table S4. BEAST priors sensitivity test. Comparison of time of divergence at 40 nodes selected across different parts of the Mycalesina tree, using different tree-shape (birth-death, yule, calibrated-yule) and clock (normal and uniform) priors. (XLSX 29 kb) Additional file 6: Table S5. Geographic connectivity matrix and the *a priori* dispersal probability constraints used for the constrained dispersal-extinction-cladogenesis (DEC) analysis. (DOCX 14 kb)

## Abbreviations

Mya: Million years ago
ML: Maximum likelihood
BI: Bayesian Inference
PP: Posterior probabilities
MCC: Maximum Clade Credibility
HPD: Highest posterior density
ESS: Effective sample sizes
MCMC: Markov Chain Monte Carlo
BIC: Bayesian Information Criterion
