# Supplementary material for: Systematics and historical biogeography of the old world butterfly subtribe Mycalesina (Lepidoptera: Nymphalidae: Satyrinae)
Source: BMC Evol Biol. 2015 Aug 20;15:167. doi: 10.1186/s12862-015-0449-3 (PMC4545879; doi:10.1186/s12862-015-0449-3)
Supplement: Additional file 2: Table S2. — The PartitionFinder estimated best-fit partitioning schemes and models of molecular evolution of Mycalesina data matrix used in the RaxML tree estimation analysis. The best model was selected under a Bayesian Information Criterion (BIC). (DOCX 20 kb) [file 12862_2015_449_MOESM2_ESM.docx]

**Additional file 2: Table S2. The best-fit partitioning schemes and models of molecular evolution of Mycalesina data matrix.** The best model was selected under a Bayesian Information Criterion (BIC) according to our PartintionFinder analysis

**All models**

| **Branch lengths** | linked |
| --- | --- |
| **Candidate models** | GTR+I, GTR+G |
| **Model selection** | BIC |
| **Search algorithm** | greedy |

**BEST PARTITIONING SCHEME**

| **Scheme Name** | step_19 |
| --- | --- |
| **Scheme lnL** | - 131016.276546 |
| **Scheme BIC** | 266571.983049 |
| **Number of parameters** | 507 |
| **Number of sites** | 7735 |
| **Number of subsets** | 11 |

| **Subset** | **Best Model** | **Subset Partitions** | **Subset Sites** |
| --- | --- | --- | --- |
| 1 | GTR+I+G | COI_1 | 1-1475\3 |
| 2 | GTR+I+G | COI_2 | 1-1475\3 |
| 3 | GTR+I+G | COI_3 | 1-1475\3 |
| 4 | GTR+I+G | Arginine_3, Efa_1, Wg_1 | 1476-2715\3, 4435-4846\3, 4849-5442\3 |
| 5 | GTR+I+G | Arginine_1, Efa_2, GAPDH_2, MAD_2, RpS5_2, Wg_2 | 1477-2715\3, 2717-3406\3, 3819-4434\3, 4436-4846\3, 4847-5442\3, 6294-7025\3 |
| 6 | GTR+I+G | Arginine_2, Efa_3, GAPDH_3, MAD_3, RpS5_3, Wg_3 | 1478-2715\3, 2718-3406\3, 3820-4434\3, 4437-4846\3, 4848-5442\3, 6295-7025\3 |
| 7 | GTR+I+G | GAPDH_1, IDH_2, RpS2_2, RpS5_1 | 2716-3406\3, 3408-3817\3, 3818-4434\3, 7027-7735\3 |
| 8 | GTR+I+G | GAPDH_1, IDH_2, RpS2_2, RpS5_1 | 3407-3817\3, 3409-3817\3 |
| 9 | GTR+I+G | CAD_1, MAD_1 | 5443-6292\3, 6293-7025\3 |
| 10 | GTR+I+G | CAD_2, IDH_3 | 5444-6292\3, 7028-7735\3 |
| 11 | GTR+I+G | CAD_3, IDH_1 | 5445-6292\3, 7026-7735\3 |
|  |  |  |  |

GTR+G = General Time Reversible plus Gamma (model 55)

SYM+G = Symmetrical Model plus Gamma (model 51)

GTR+G = General Time Reversible plus Gamma (model 55)

TrNef+G = Equal-frequency Tamura-Nei plus Gamma (model 19)

K80+G = Kimura 2-parameter plus Gamma (model 11)

Scheme Description in PartitionFinder format

Scheme_step_19 = (CO1_1) (CO1_2) (CO1_3) (Arginine_3, Efa_1, Wg_1) (Arginine_1, Efa_2, GAPDH_2, MAD_2, RpS5_2, Wg_2) (Arginine_2, Efa_3, GAPDH_3, MAD_3, RpS5_3, Wg_3) (GAPDH_1, IDH_2, RpS2_2, RpS5_1) (RpS2_1, RpS2_3) (CAD_1, MAD_1) (CAD_2, IDH_3) (CAD_3, IDH_1);

**RaxML-style partition definitions**

DNA, p1 = 1-1475\3

DNA, p2 = 2-1475\3

DNA, p3 = 3-1475\3

DNA, p4 = 1476-2715\3, 4435-4846\3, 4849-5442\3

DNA, p5 = 1477-2715\3, 2717-3406\3, 3819-4434\3, 4436-4846\3, 4847-5442\3, 6294-7025\3

DNA, p6 = 1478-2715\3, 2718-3406\3, 3820-4434\3, 4437-4846\3, 4848-5442\3, 6295-7025\3

DNA, p7 = 2716-3406\3, 3408-3817\3, 3818-4434\3, 7027-7735\3

DNA, p8 = 3407-3817\3, 3409-3817\3

DNA, p9 = 5443-6292\3, 6293-7025\3

DNA, p10 = 5444-6292\3, 7028-7735\3

DNA, p11 = 5445-6292\3, 7026-7735\3
