# Supplementary figures and images for: Systematics and historical biogeography of the old world butterfly subtribe Mycalesina (Lepidoptera: Nymphalidae: Satyrinae)
Source: BMC Evol Biol. 2015 Aug 20;15:167. doi: 10.1186/s12862-015-0449-3 (PMC4545879; doi:10.1186/s12862-015-0449-3)

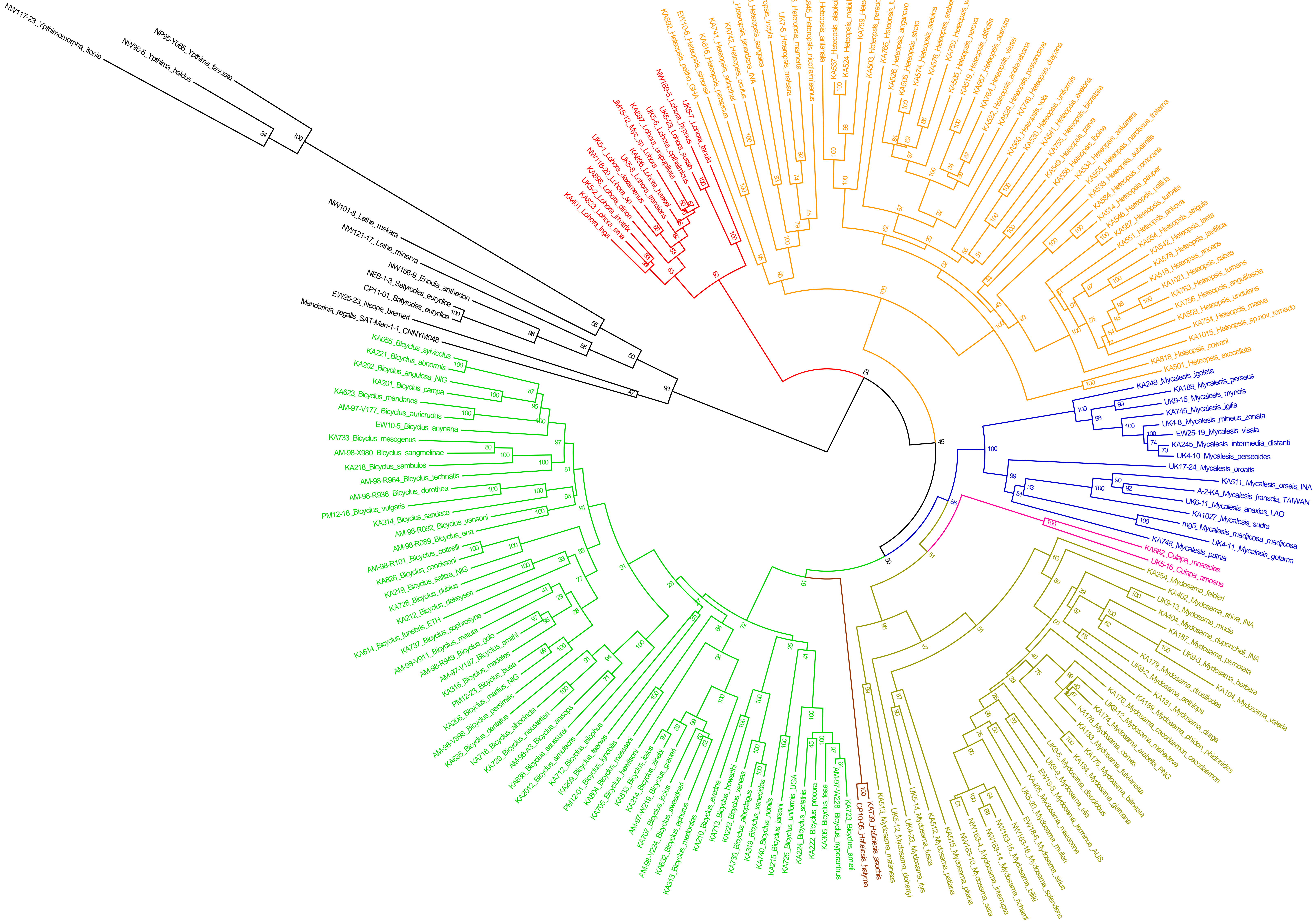

Supplement: Additional file 4: Figure S1. — Phylogenetic relationships of the different genera within the subtribe Mycalesina estimated using RaxML maximum likelihood method. A consensus phylogeny is shown using the combined 10 genes dataset. The numbers besides the nodes are the bootstrap support values. (PDF 82 kb) [file 12862_2015_449_MOESM4_ESM.pdf]
