# Supplementary material for: Systematics and historical biogeography of the old world butterfly subtribe Mycalesina (Lepidoptera: Nymphalidae: Satyrinae)
Source: BMC Evol Biol. 2015 Aug 20;15:167. doi: 10.1186/s12862-015-0449-3 (PMC4545879; doi:10.1186/s12862-015-0449-3)
Supplement: Additional file 6: Table S5. — Geographic connectivity matrix and the a priori dispersal probability constraints used for the constrained dispersal-extinction-cladogenesis (DEC) analysis. (DOCX 14 kb) [file 12862_2015_449_MOESM6_ESM.docx]

Additional file 6; Table S5. The connectivity matrix for the DEC analyses. The set probabilities of a mycalesine to disperse from one geographic region to another.

|  |  | **Africa** | **Madagascar** | **Asia** | **Wallacea** | **Australasian Region** |
| --- | --- | --- | --- | --- | --- | --- |
|  |  |  |  |  |  |  |
| **Africa** |  | 1 | 1 | 1 | 0.00001 | 0.00001 |
| **Madagascar** |  | 1 | 1 | 0.1 | 0.001 | 0.00001 |
| **Asia** |  | 1 | 0.1 | 1 | 1 | 0.001 |
| **Wallacea** |  | 0.00001 | 0.001 | 1 | 1 | 1 |
| **Australasian Region** |  | 0.00001 | 0.00001 | 0.001 | 1 | 1 |
|  |  |  |  |  |  |  |
